# Endoscopic retrograde cholangiopancreatography treatment for biliary dilatation in pediatric patients: a retrospective multicenter study

**DOI:** 10.1186/s12887-025-06047-z

**Published:** 2025-08-28

**Authors:** Sheng Ding, Jing-Qing Zeng, Zhao-Hui Deng, Tian-Ao Zhang, Biao Gong

**Affiliations:** 1 Department of Gastroenterology, Shanghai Children’s Medical Center, Shanghai Jiao Tong University School of Medicine, Shanghai, 200127 China; 2 Department of Digestive Diseases, Shanghai Shuguang Hospital, Shanghai University of Traditional Chinese Medicine, Shanghai, 201203 China

**Keywords:** Biliary dilatation, Endoscopic retrograde cholangiopancreatography, Treatment, Limitation

## Abstract

**Background:**

The main treatments for biliary dilatation (BD) are surgical resection and choledochojejunostomy. However, various factors lead to the postponement of early surgical intervention in pediatric BD patients. This study aimed to analyze the effectiveness and limitations of endoscopic retrograde cholangiopancreatography (ERCP) in BD pediatric patients.

**Methods:**

Data on patients with BD treated at two centers from June 2018 to June 2022 were retrospectively collected. Patients were categorized into five groups according to Todani classification. Clinical features, ERCP processes, ERCP-related complications, ERCP effectiveness and its limitations were reviewed.

**Results:**

A cohort of 77 symptomatic BD patients was evaluated, with pancreaticobiliary malformation (PBM) identified in 68.8% of cases. These patients underwent 142 hospitalizations, including 96 involving ERCP and 46 without ERCP. ERCP was performed in 74.0% (57/77) of BD patients, with a median follow-up of 26 months (range: 2–48 months), achieving an effectiveness rate of 75.4%. ERCP-related complications included post-ERCP pancreatitis (20.8%), postoperative infections (6.3%), and bleeding (2.1%). All complications were mild and managed conservatively. Further stratified analysis revealed that patients under 36 months of age at onset (*P* = 0.001) and those with elevated liver enzymes (*P* = 0.049) did not respond well to ERCP. The proportion of jaundice (*P* = 0.006) and biliary obstruction (*P* = 0.013) was significantly higher in the group with an onset age of ≤ 36 months compared to the group with an onset age > 36 months.

**Conclusions:**

ERCP is suitable for BD pediatric patients with mild and acceptable complications. Careful consideration is required when deciding to perform the procedure, as therapeutic outcomes are limited in patients with an onset age under 36 months.

## Introduction

Biliary dilatation (BD), also known as congenital biliary dilatation or choledochal cysts, is a condition characterized by the abnormal dilatation of segments of the intrahepatic and extrahepatic bile ducts. This condition primarily manifests in childhood and infancy, with significantly higher incidence rates in Eastern populations than in Western ones. The incidence rate is estimated at 1 in 1,000 live births in Japan and Southeast Asia, sharply contrasting with the rates of approximately 1 in 100,000 to 1 in 150,000 in Western countries [1, 2]. A significant long-term complication of BD is the development of biliary tract malignancies, primarily cholangiocarcinoma or gallbladder carcinoma. Therefore, early surgical intervention is strongly recommended upon diagnosis to reduce these risks [3].

However, some children with BD cannot undergo immediate surgery due to conditions such as fruste biliary dilatation, young age, or insufficient parental consent. Reports have highlighted the use of endoscopic retrograde cholangiopancreatography (ERCP) in treating BD [4–7]. However, these studies often involve small sample sizes, particularly in pediatric populations, limiting their scope. This study aimed to assess the safety and efficacy of ERCP in the treatment of BD while also identifying its limitations.

## Patients and methods

### Patients

BD cases were retrospectively collected from patients hospitalized at two centers between June 2013 and June 2022. The inclusion criteria were as follows: (1) Age ≤ 18 years at onset; (2) Diagnosis of BD without prior surgical intervention. The exclusion criteria were as follows: (1) Secondary biliary dilatation from causes such as bile duct stones, tumors, or post cholecystectomy changes; (2) Incomplete case or ERCP information. During this process, we analyzed the clinical features of different BD types, examined the efficacy and complications of BD patients treated with ERCP, and summarized the effectiveness and safety of ERCP for BD patients.

### Definitions

#### Definitions of BD

BD was defined on the basis of the 2016 Japanese Clinical Practice Guidelines for congenital biliary dilatation published by the Japanese Study Group on Congenital Biliary Dilatation (JSCBD) [8]. The bile duct diameter was measured via non-pressure methods such as ultrasound (US), computed tomography (CT), and magnetic resonance imaging (MRI), and the results were compared with the bile duct diameter data published by the Japanese Study Group on Pancreaticobiliary Maljunction (JSGPBM) [9]. BD was diagnosed when the diameter exceeded the upper limit, and secondary causes were excluded. The Todani classification is based on the morphology of the intrahepatic and extrahepatic bile ducts [10].

#### Definitions of Post-ERCP pancreatitis

Post-ERCP pancreatitis (PEP) is defined as the development of new-onset persistent upper abdominal pain lasting more than 24 h after ERCP, along with elevated serum amylase or lipase levels, typically exceeding three times the upper limit of normal [11].

#### Definitions of the effectiveness of ERCP for BD patients

The effectiveness of ERCP treatment was defined as the absence of BD-related clinical symptoms from up to two ERCP interventions until the end of the follow-up period. Some patients may require multiple ERCP procedures to achieve the initial therapeutic goals. For example, factors such as young age, a limited duodenal space, or papillary edema may prevent the completion of procedures such as stone extraction, dilatation, or sphincterotomy in a single session, necessitating a second ERCP. If a patient undergoes three or more ERCP procedures, these procedures are no longer considered part of the original treatment plan but rather interventions for recurrent symptoms following the initial ERCP. These cases suggest that ERCP is ineffective or that the patient does not respond to the procedure.

### Instruments and materials

The main instruments and materials used in ERCP include duodenoscopes (1) Olympus JF260 (tip diameter: 12.6 mm, insertion diameter: 11.3 mm, working channel diameter: 3.7 mm) and (2) Olympus JF240 (tip diameter: 12.6 mm, insertion diameter: 11.0 mm, working channel diameter: 3.2 mm), along with guidewires, sphincterotomes, dilating catheters, stone retrieval balloons, stone retrieval baskets, bile duct stents, and pancreatic duct stents, etc.

### Indications for ERCP in BD patients and ERCP procedures

Indications for ERCP in cases of biliary dilatation include cholangitis, bile duct stones, biliary pancreatitis, obstructive jaundice, and pancreatic duct protein plugs. At these two centers, ERCPs are performed by 3 and 2 experienced endoscopists, respectively. None of the patients received nonsteroidal anti-inflammatory drugs (NSAIDs) prior to the ERCP procedure. The main ERCP procedures are endoscopic sphincterotomy (EST), endoscopic pancreatic sphincterotomy (EPST), endoscopic papilla balloon dilatation (EPBD), stone removal, endoscopic retrograde biliary drainage (ERBD), endoscopic retrograde pancreatitis (ERPD), endoscopic nasal pancreatic drainage (ENPD), and endoscopic nasal biliary drainage (ENBD).

### Statistical analysis

Statistical analysis was conducted via SPSS 20.0 software. Categorical data and corresponding percentages are expressed as n(%). Comparisons between groups for categorical data were performed via the chi-square test. Skewed continuous data are presented as the median (M), 25th percentile (P25), and 75th percentile (P75) and were formatted as M (P25, P75). An independent t test was used to compare continuous variables between the two groups. The relationship between ERCP effectiveness and month age at disease onset was analyzed using receiver operating characteristic curves (ROC).

### Declaration of generative AI and AI-assisted technologies in the writing process

During the preparation of this work, the authors used chatGPT and Deepseek to improve the spelling, grammar, clarity, conciseness and overall readability of the text provided. After using these tools, the authors reviewed and edited the content as needed and take full responsibility for the content of the publication.

## Results

### General clinical characteristics

A total of 77 children with biliary dilatation (BD) were included in the study, comprising 25 males and 52 females, with females accounting for 67.5% of the cohort. The median age at BD onset was 35 months (range: 0–155 months). The primary clinical symptoms included abdominal pain (61/77, 79.2%) and vomiting (43/77, 55.8%). Laboratory findings revealed elevated liver enzymes in 66.2% of the patients (51/77) and elevated pancreatic enzymes in 55.8% (43/77). Among the 77 BD cases, Todani types I (36/77) and IV (34/77) were the most prevalent, whereas types II, III, and V were less common. The incidence rates of bile duct stones and pancreatitis in BD patients were identical, occurring in 63.6% of cases for both conditions. Notably, no cases of biliary tract cancer or gallbladder carcinoma were observed. Additionally, 68.8% of the children with BD (53/77) exhibited pancreaticobiliary malformation (PBM). See Table 1.

### Relationships between the clinical characteristics of BD patients and the Todani classification

Among the 77 patients with biliary dilatation (BD), there were 36 cases of type I BD, 1 case of type II BD, 1 case of type III BD, 34 cases of type IV BD, and 5 cases of type V BD. Types I and IV BD constituted the majority of cases, accounting for 90.9% (70/77) of the total. Given the limited number of cases for types II, III, and V, the statistical analysis focused on comparing types I and IV. The analysis revealed no significant differences between types I and IV regarding sex, age of onset, proportion of ERCP treatments, most clinical symptoms, elevated pancreatic enzymes, or inflammatory markers. However, jaundice, elevated liver enzymes, hyperbilirubinemia, and bile duct stones were more frequently observed in type IV patients than in type I patients. Hyperbilirubinemia exhibited the most significant difference (*P* = 0.009), followed by jaundice (*P* = 0.01), bile duct stones (*P* = 0.034), and elevated liver enzymes (*P* = 0.034). Additionally, the incidence rate of PBM was greater in patients with type IV BD (*P* = 0.041). See Table 2.

### Effectiveness, safety and limitations of ERCP in BD patients

A total of 57 patients with BD underwent ERCP. The highest proportion of these patients was in the 3–10 years age group, comprising 57.8% (33/57) of the cohort. Among the different Todani classification types, patients with type I BD and type IV BD received ERCP at similar rates, with 75.0% (27/36) and 82.3% (28/34), respectively. ERCP was also performed on patients with type II and type III BD, with one case each. The most commonly performed procedures during ERCP for BD children include endoscopic sphincterotomy (EST), endoscopic pancreatic sphincterotomy (EPST), endoscopic bile duct stone removal, and endoscopic bile duct stent placement. The overall complication rate associated with ERCP in these BD patients was 29.2% across 96 procedures, with post-ERCP pancreatitis (PEP) being the most common complication, occurring in 20.8% of cases. Postoperative infections occurred in 6.3% of cases, with cholangitis and cholecystitis each accounting for 2.1% of the patients. Additionally, postoperative bleeding was noted in 2.1% of cases. Notably, there were no reported instances of intestinal perforation. All complications were mild and were successfully managed with conservative treatment. The median follow-up period was 26 months, during which the overall effectiveness rate of the ERCP intervention was 75.4%, with no patients lost to follow-up. During this follow-up period, 50.9% (29/57) of the ERCP patients underwent surgical interventions, all of which were Roux-en-Y procedures. Follow-up data for patients who underwent surgical procedures were not included in this study. See Table 3.

57 patients underwent a total of 96 ERCP procedure, with an average of 1.7 procedures per patient. Among these, 38 children had a single ERCP, 5 had two ERCP procedures, and 14 had three or more ERCP procedures. Clinical remission was achieved in 75.4% (43/57) of patients after two or fewer ERCPs, which represented 50% (48/96) of the total procedures performed, with an average of 1.1 procedures per patient. The remaining 14 patients (24.6%) required the other 50% (48/96) of ERCP procedures, averaging 3.4 procedures per patient. Patients were categorized according to the number of ERCP procedures they underwent. Patients who underwent two or fewer ERCPs without recurrence were classified as sensitive to ERCP treatment, whereas those requiring three or more ERCPs were classified as insensitive. A thorough analysis was conducted between these two groups, considering variables such as age at onset, age at the time of ERCP, sex, BD classifications, PBM, clinical manifestations, ERCP-related complications, and specific ERCP techniques. The analysis revealed a statistically significant difference between the two groups in terms of age at onset (*p* = 0.001) and a marginally significant difference in elevated liver enzyme levels (*p* = 0.049). Notably, patients with an onset age of 36 months or younger were significantly more likely to require three or more ERCP procedures, suggesting that a younger onset age is associated with a greater likelihood of insensitivity to ERCP treatment. See Table 4.

**Fig. 1:**
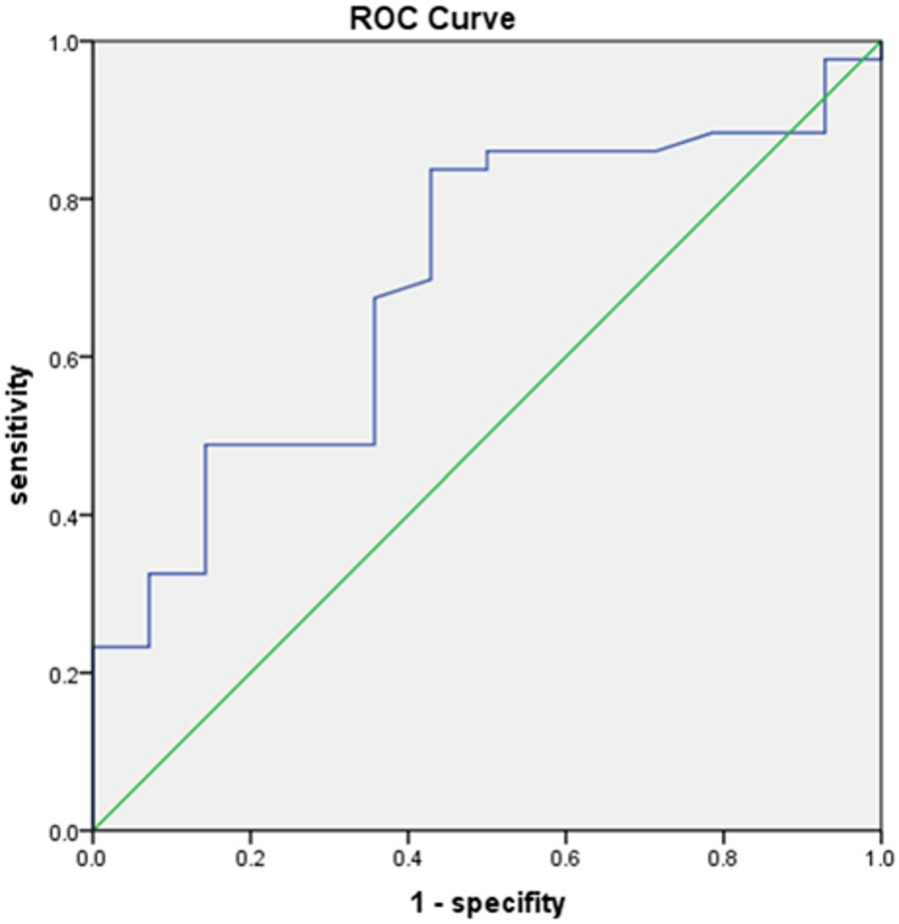
ROC curve between month age at disease onset and ERCP effectiveness. AUC = 0.786, *P* = 0.029. ROC: Receiver operating characteristic, AUC: Area under curve

Receiver operating characteristic (ROC) curves were used to define a more precise onset age, to further analyze the relationship between the onset age and the effectiveness of ERCP. ROC analysis indicated that the area under the curve (AUC) was 0.786, with a P-value of 0.029 (see Fig. 1), suggesting statistical significance. The cutoff value for onset age was calculated using the Youden index, which was found to be 38.5 months, with a sensitivity of 78.8% and specificity of 71.4%. The onset age of 38.5 months, determined by ROC analysis, was close to the value of 36 months identified by univariate analysis.

Given the significant statistical difference in onset age observed in the univariate analysis (*P* = 0.001), we aimed to further investigate the differences between various onset ages. The analysis continued with 57 patients who underwent ERCP, categorized based on whether their onset age exceeded 36 months. Demographic characteristics, clinical manifestations, BD Todani classification, laboratory tests, and ERCP-related events were compared. The group with an onset age of ≤ 36 months had significantly higher proportions of jaundice (*P* = 0.006) and clay-colored stools (*P* = 0.013) compared to those with an onset age > 36 months. No significant differences were found in other aspects. See Table 5.

## Discussion

BD is primarily detected in childhood, with an incidence of approximately 20%−25% in adults [12]. Previous studies suggest that the typical clinical presentation in children includes a triad of symptoms, namely, abdominal pain, a palpable abdominal mass, and jaundice, with approximately 85% of pediatric patients exhibiting at least two of these symptoms [1, 13]. As shown in this study, abdominal pain, vomiting, and jaundice were the most common clinical symptoms, accounting for 93.8% of the patients. Other relatively common symptoms included fever and pale stools, while a palpable abdominal mass was rare, accounting for only 2.6% of the symptoms. Notably, a study involving 55 children with BD also revealed no persistent abdominal masses [4]. This finding indicates that an abdominal mass cannot be solely used as a diagnostic or exclusion criterion for BD. Additionally, there is a difference in the incidence of BD between sexes. The Japanese Study Group on Congenital Biliary Dilatation (JSCBD) reported that the male-to-female ratio is approximately 1:3 [3]. Similarly, Angelis et al. reported comparable findings in a study of 28 pediatric BD patients [13]. In our study, the male-to-female ratio was 1:2.1, which closely aligns with the conclusions of previous studies. However, the reasons for the sex disparity in BD incidence remain unclear. Some studies suggest that Todani type I is more common in children, whereas Todani type IV is more frequently observed in adults [14]. In our study, we observed a slightly greater prevalence of type I BD than type IV BD in pediatric BD cases, though the difference was not statistically significant.

Tsuchiya et al. [4] reported that elevated liver and pancreatic enzymes are the most common laboratory findings in pediatric BD patients. These findings align with our findings, where elevated liver and pancreatic enzymes were the most frequent abnormalities, followed by hyperbilirubinemia and elevated inflammatory markers. The liver and pancreatic damage observed in BD patients is associated primarily with bile stasis or impaired bile drainage and pancreaticobiliary malformation (PBM). We observed that the proportion of children with type IV BD exhibiting hyperbilirubinemia, elevated liver enzymes, and jaundice was significantly greater than that of those with type I BD. This may be attributed to the greater prevalence of bile stasis, intrahepatic bile duct stones, and cholangitis in type IV BD cases [14], which are more likely to lead to liver dysfunction, such as elevated bilirubin and liver enzyme levels.

The view that PBM is considered a necessary condition for diagnosing BD has not been widely accepted in clinical practice in certain regions, including China [1]. In 2017, JSCBD proposed that, considering the hypothesized role of PBM in BD pathophysiology, PBM should be regarded as a necessary diagnostic criterion for BD [3]. In that same year, the Committee on Diagnostic Criteria of JSGPBM published a multicenter, 13-year retrospective study involving 317 children with PBM, which found that 92.4% of these cases were associated with BD [15]. In contrast, a 2019 retrospective study from our center involving 75 children with PBM showed that 61.3% (46/75) of these patients had concurrent BD [16], a significantly lower proportion than the 92.4% reported by JSGPBM. In addition, in 2024, a clinical study from China reported treatment options for 68% PBM patients without concurrent BD [17]. Additionally, studies from other centers in Europe and beyond have reported that the rate of BD complicating PBM ranges from 50 to 80% [1, 12, 14, 18, 19]. In this study, the overall prevalence of BD complicating PBM was 68.8%, with PBM present in 75.7% (53/70) of patients with type I and type IV BD. Moreover, PBM was not observed in cases of type II, type III, or type V BD (Caroli disease) in this study, which may suggest that the pathogenesis of type II, III, and V BD differs to some extent from that of type I and IV BD. Our findings suggest that while a substantial proportion of BD patients may have concurrent PBM, there is insufficient evidence to support PBM as a necessary condition for BD. Caroli disease (type V BD) has previously been associated with mutations in the polycystic kidney and hepatic disease 1 (*PKHD1*) gene [20]. Given the rarity of type II and type III BDs, research on these types is limited.

Reports on the application of ERCP for managing BD in pediatric patients are sparse. Angelis et al. [13] described ERCP in 28 children with BD prior to surgical intervention in 2012, highlighting its safety and adaptability in this population. They noted that ERCP effectively delineates anatomical structures and facilitates appropriate surgical planning, including timing and technique selection. Subsequent case reports have discussed ERCP treatment for specific types of BD, such as type III BD [5, 21, 22]. Compared with these earlier studies, which involved fewer cases and focused on a narrower range of BD types, our research included 57 children aged 0-155 months who underwent ERCP. This study included all Todani classifications of BD and encompasses a much broader age range of children. Our findings further support the safety and efficacy of ERCP for managing pediatric BD.

The incidence of PEP in this study was 20.8%, significantly higher than the overall incidence of 10.2% reported in a 2023 review of 145 randomized controlled trials [23]. Another study conducted by our center, published in 2021, reported a 20.7% (19/92) incidence of PEP, focusing on a spectrum of pediatric diseases, including chronic pancreatitis, pancreaticobiliary malformation, pancreatic divisum, and pancreatic pseudocyst [24]. Similar to our findings, Qian et al. [17] reported a 15.1% proportion of PEP in a 2024 study focused on pediatric patients. Compared with adults, it is evident that larger pediatric pancreaticobiliary centers in China have reported a higher incidence of PEP. Several factors could contribute to this higher rate: (1) The disease spectrum in children differs from that in adults, with pancreatic conditions being the primary indication for ERCP in pediatric patients. Consequently, the incidence of PEP is relatively high in children. This study required detailed visualization of both the bile duct and the pancreatic duct, necessitating repeated contrast medium injections. In some cases, guidewires are needed to access the pancreatic duct, which may increase the postoperative PEP rate compared with selective bile duct cannulation and contrast in adults. (2) Effective preventive measures for PEP are lacking. While nonsteroidal anti-inflammatory drugs (NSAIDs), such as indomethacin, are used for prevention in adults, there is no consensus regarding their use in children. (3) Despite the use of pain scoring tools, children’s descriptions of pain tend to be less precise than those of adults, which may lead to misdiagnosis of some cases of hyperamylasemia as PEP. Nonetheless, no severe cases or fatalities were observed among the PEP patients in this study, further confirming the safety of ERCP in pediatric BD patients.

Not all patients with BD are likely to benefit from ERCP treatment, a consideration that has not been highlighted in previous studies. In this study, the majority of patients (75.4%) experienced effective relief of clinical symptoms after two or fewer ERCP interventions. In contrast, a minority of patients (24.6%) who underwent multiple ERCP procedures (maximum of 5 procedures) continued to experience recurrent symptoms. This suggests that, for some patients, increasing the number of ERCP interventions may not lead to significant clinical benefit. Younger patients (≤ 36 months) may face greater challenges in achieving clinical remission through ERCP alone, especially those without elevated liver enzymes. Further analysis revealed that jaundice and clay-colored stools were significantly more common in patients with an onset age of ≤ 36 months compared to those with an onset age > 36 months. Interestingly, there was no significant difference between the two groups in terms of hyperbilirubinemia. The presence of facial jaundice and clay-colored stools requires higher serum bilirubin levels and biliary obstruction. This suggests a difference in bilirubin levels between the two groups, with patients ≤ 36 months more likely to exhibit elevated serum bilirubin levels and biliary obstruction symptoms. we found that the proportion of patients with elevated bilirubin levels was significantly higher in Todani type IV compared to type I (*P* = 0.009, see Table 2). When jaundice differences were observed across onset age groups, the BD Todani classification was also examined. Although the proportion of type IV was higher in the ≤ 36-month group compared to the > 36-month group (58.1% vs. 38.5%), the difference was not statistically significant. This suggests that the BD classification does not have a significant role, and the cause of higher bilirubin levels in the younger onset age group remains unclear and requires further investigation. These findings suggest that a more nuanced approach is necessary when considering the need for more aggressive and expensive treatment strategies in this age group. Whether recurrence after two ERCP procedures can serve as a criterion for determining the suitability of ERCP treatment remains a topic for further research and discussion.

This study had several limitations. It was retrospective, and although the average follow-up period was relatively long (26 months), the follow-up duration for each patient varied. This variability may introduce bias when using clinical recurrence after ERCP treatment as an endpoint. Additionally, this study found that patients with an onset age of less than 36 months were less responsive to ERCP. However, due to the nature of the study, direct evidence could not be established. Additionally, this study has not examined the prognostic outcomes of enrolled patients undergoing surgical intervention, and therapeutic benefits need to be compared with surgical cohorts in further studies.

In conclusion, this study comprehensively examined BD in pediatric patients, emphasizing key clinical features and evaluating the efficacy of ERCP as a treatment option. It typically presents with abdominal pain, vomiting, and jaundice, with Todani types I and IV being the most common. Our findings indicate that ERCP is a safe and effective method for managing BD. Although PEP occurs much more frequently in pediatric patients than in adults, ERCP successfully alleviates symptoms in most cases. However, younger patients, particularly those under 36 months of age at onset, may require additional treatments owing to their reduced responsiveness to ERCP.

**Table 1:** General clinical characteristics of children with biliary dilatation

Category	*N* = 77	Category	*N* = 77
**Gender (Male: Female)**	25:52	Hyperbilirubinemia	32 (41.6)
**Age of Onset (Months)**,**M(P25**,** P75)**	35 (18, 70)	**TODANI Classification n (%)**	
**Clinical Symptoms**,** n (%)**		Type I	36 (46.8)
Abdominal pain	61 (79.2)	Type II	1 (1.3)
Vomiting	43 (55.8)	Type III	1 (1.3)
Jaundice	22 (28.6)	Type IV	34 (44.2)
Pale stool	13 (16.9)	Type V	5 (6.5)
Abdominal mass	2 (2.6)	**Complications n (%)**	
**Laboratory Findings**,** n (%)**		Bile duct stones	49 (63.6)
Elevated liver enzymes	51 (66.2)	Pancreatitis	49 (63.6)
Elevated pancreatic enzymes	43 (55.8)	PBM	53 (68.8)
Elevated inflammatory markers	27 (35.1)	**ERCP n (%)**	57 (74.0)

ERCP: Endoscopic Retrograde Cholangiopancreatography

The use of bold text in certain items in the table is intended to distinguish between main categories and subcategories, with the main categories in bold. From my perspective, this enhances the readability of the table. If you believe this affects the aesthetic of the format, it can be adjusted. I have attempted to change it to non-bold text but was unsuccessful

**Table 2:** Correlation analysis of clinical characteristics and biliary dilatation Todani classification in children

Todani Type	I (*n* = 36)	II (*n* = 1)	III (*n* = 1)	IV (*n* = 34)	V (*n* = 5)	*P*-value^a^
**Gender(Male/Female)**	11/25	1/0	0/1	9/25	4/1	0.705
**Age of Onset (Months)**						0.977
0 < M ≤ 12	4	0	0	4	1	
12 < M ≤ 36	16	1	0	14	1	
36 < M ≤ 120	14	0	1	15	3	
120 < Y	2	0	0	1	0	
**Clinical Symptoms**,** n (%)**						
Abdominal pain	28	1	1	29	2	0.419
Vomiting	20	0	0	21	2	0.598
Jaundice	5	0	1	14	2	**0.010**
Fever	7	0	1	8	0	0.677
Pale stool	6	0	0	6	1	0.913
Abdominal mass	1	0	0	1	0	1.000
**Laboratory Findings**,** n (%)**						
Elevated liver enzymes	20	0	1	27	3	**0.034**
Elevated pancreatic enzymes	21	0	1	21	0	0.770
Elevated inflammatory markers	13	0	1	11	2	0.741
Hyperbilirubinemia	10	0	1	20	1	**0.009**
**Complications**						
Bile duct stones	20	0	1	27	1	**0.034**
Pancreatitis	23	0	1	24	1	0.551
PBM	23	1	0	29	0	**0.041**
**ERCP**	27	1	1	28	0	0.454

a: Comparative analysis between Todani Type I and Type IV, ERCP: Endoscopic Retrograde Cholangiopancreatography, PBM: Pancreaticobiliary Malformation

**Table 3:** ERCP treatment for biliary dilatation patients

Category		*n* = 57	Category	*n* = 57	
**Gender (Male: Female)**		12:45	Stone extraction	28	
**Age (Years)**			EPBD	10	
0 < Y ≤ 1		3	Bougie Dilatation	5	
1 < Y ≤ 3		15	**Number of ERCP Procedures**		
3 < Y ≤ 10		33	1	38	
10 < Y		6	2	5	
**Todani Classification**			3	10	
Type I		27	4	2	
Type II		1	5	2	
Type III		1	**ERCP Complications**,** n (%)**		
Type IV		28	PEP	20(20.8)	
**ERCP Procedure**			Cholangitis/Cholecystitis	2 (2.1)	
ERCP		28	Bleeding	2 (2.1)	
ERC		8	Infection	4 (4.2)	
ERP		2	Perforation	0(0)	
EST/EPST		52	**Follow-up**		
ERBD	25		Follow-up duration (months)	26 (2-106)	
ERPD	10		Lost to follow-up, n	0	
ENBD	4		Effectiveness rate, %	75.4	
ENPD	0		**Subsequent surgical intervention**, n (%)	29 (50.9%)	

ERCP: Endoscopic Retrograde Cholangiopancreatography, ERC: Endoscopic Retrograde Cholangiography, ERP: Endoscopic Retrograde Pancreatography, EST: Endoscopic Sphincterotomy, EPST: Endoscopic Pancreatic Sphincterotomy, EPBD: Endoscopic Papilla Balloon Dilation, ERBD: Endoscopic Retrograde Biliary Drainage, ERPD: Endoscopic Retrograde Pancreatic Drainage, ENPD: Endoscopic Nasal Pancreatic Drainage, ENBD: Endoscopic Nasal Biliary Drainage, PEP: Post-ERCP Pancreatitis

**Table 4:** Effectiveness of ERCP in BD patients continued

Terms	Variables	Effective (*n* = 43)	Ineffective (*n* = 14)	*P*-value
**Demographics**	Gender (Male/Female)	8/35	4/10	0.463
	Age at onset ≤ 36/>36 months	10/20	21/6	**0.001**
**Clinical Presentation**	Abdominal pain	30	12	0.629
	Vomiting	27	10	0.749
	Fever	9	5	0.297
**Laboratory Findings**	Elevated CRP/WBC	13	7	0.209
	Elevated liver enzymes	31	6	**0.049**
	Elevated pancreatic enzymes	29	8	0.530
	Hyperbilirubinemia	18	7	0.505
**BD Complications**	Bile duct stones	27	11	0.343
	Pancreatitis	31	11	0.739
	PBM	36	12	1.000
**ERCP Parameters**	Age at ERCP ≤ 36/>36 months	14/29	4/10	1.000
	ERCP Complications	16	7	0.397
**ERCP Interventional Techniques**	EST	16	7	0.200
	EPST	19	5	0.454
	Papillary dilatation	7	3	0.694
	ENBD	4	0	0.563
	Biliary stenting	20	5	0.479
	Pancreatic stenting	7	3	0.694
	Stone extraction	21	7	1.000
	Biliary Dilatation	3	2	0.587
	Cholangiopancreatography	22	6	0.589

CRP: C-reactive Protein; WBC: White Blood Cell; BD: Biliary Dilatation; PBM: Pancreaticobiliary Malformation; ERCP: Endoscopic Retrograde Cholangiopancreatography; EPST: Endoscopic Pancreatic Sphincterotomy; ENBD: Endoscopic Nasal Biliary Drainage

**Table 5:** Comparison of clinical characteristics between biliary dilatation patients with onset age ≤ 36 months vs. >36 months

Category	Variable	≤ 36 Months (*n* = 31)	> 36 Months (*n* = 26)	*P*-value
**Demographics**	Sex (Male/Female)	9/22	3/23	0.107
**Clinical Features**	Vomiting	23	16	0.306
	Fever	8	6	0.812
	Clay-colored stool	9	1	**0.013**
	Jaundice	14	3	**0.006**
	Abdominal pain	24	26	0.010^a^
**Laboratory Tests**	Elevated liver enzymes	23	15	0.188
	Elevated inflammatory markers	12	8	0.532
	Elevated pancreatic enzymes	19	18	0.532
	Hyperbilirubinemia	16	9	0.198
**BD Classifications**	Todani-I	13	16	0.140
	Todani-IV	18	10	
**ERCP Related**	ERCP complications	11	13	0.269
	PEP	8	13	0.059
	Cholangitis	1	1	0.899
	Bleeding	2	0	0.187

BD: Biliary Dilatation; ERCP: Endoscopic Retrograde Cholangiopancreatography; PEP: Post ERCP Pancreatitis

## Data Availability

The datasets generated and/or analyzed during the current study are not publicly available due to the organization’s data protection requirements but are available from the corresponding author on reasonable request.

## Abbreviations

BD: Biliary dilation
ERCP: Endoscopic retrograde cholangiopancreatography
PBM: Pancreaticobiliary malformation
JSCBD: The Japanese study group on congenital biliary dilatation
US: Ultrasound
CT: Computed tomography
MRI: Magnetic resonance imaging
JSGPBM: The Japanese study group on pancreaticobiliary maljunction
PEP: Post-ERCP pancreatitis
EST: Endoscopic sphincterotomy
EPST: Endoscopic pancreatic sphincterotomy
EPBD: Endoscopic papilla balloon dilation
ERBD: Endoscopic retrograde biliary drainage
ERPD: Endoscopic retrograde pancreatic drainage
ENPD: Endoscopic nasal pancreatic drainage
ENBD: Endoscopic nasal biliary drainage
CNY: Chinese yuan
PKHD1: The polycystic kidney and hepatic disease 1 gene
NSAIDs: Nonsteroidal anti-inflammatory drugs
